# Herpes Simplex Virus Type 1 Infection of the Central Nervous System: Insights Into Proposed Interrelationships With Neurodegenerative Disorders

**DOI:** 10.3389/fncel.2019.00046

**Published:** 2019-02-26

**Authors:** Luisa F. Duarte, Mónica A. Farías, Diana M. Álvarez, Susan M. Bueno, Claudia A. Riedel, Pablo A. González

**Affiliations:** ^1^Millennium Institute on Immunology and Immunotherapy, Departamento de Genética Molecular y Microbiología, Facultad de Ciencias Biológicas, Pontificia Universidad Católica de Chile, Santiago, Chile; ^2^Millennium Institute on Immunology and Immunotherapy, Departamento de Biología Celular, Facultad de Ciencias de la Vida, Universidad Andrés Bello, Santiago, Chile

**Keywords:** herpes simplex virus, neurodegeneration, neurological disease, apoptosis, autophagy, mitochondrial damage, oxidative stress, neuroinflammation

## Abstract

Herpes simplex virus type 1 (HSV-1) is highly prevalent in humans and can reach the brain without evident clinical symptoms. Once in the central nervous system (CNS), the virus can either reside in a quiescent latent state in this tissue, or eventually actively lead to severe acute necrotizing encephalitis, which is characterized by exacerbated neuroinflammation and prolonged neuroimmune activation producing a life-threatening disease. Although HSV-1 encephalitis can be treated with antivirals that limit virus replication, neurological sequelae are common and the virus will nevertheless remain for life in the neural tissue. Importantly, there is accumulating evidence that suggests that HSV-1 infection of the brain both, in symptomatic and asymptomatic individuals could lead to neuronal damage and eventually, neurodegenerative disorders. Here, we review and discuss acute and chronic infection of particular brain regions by HSV-1 and how this may affect neuron and cognitive functions in the host. We review potential cellular and molecular mechanisms leading to neurodegeneration, such as protein aggregation, dysregulation of autophagy, oxidative cell damage and apoptosis, among others. Furthermore, we discuss the impact of HSV-1 infection on brain inflammation and its potential relationship with neurodegenerative diseases.

## Introduction

Herpes simplex virus type-1 (HSV-1) is an enveloped double-stranded DNA virus belonging to the *Herpesviridae* family, that has a genome of approximately 152 kbp encoding more than 80 different open reading frames (ORFs; Boehmer and Nimonkar, 2003). Importantly, HSV-1 is a neurotropic pathogen with a wide spectrum of clinical disorders ranging from harmless skin manifestations, such as oral and facial lesions to severe infection of the central nervous system (CNS). HSV-1 is the most common cause of sporadic encephalitis in adults, as well as the leading cause of infectious blindness in developed countries due to herpetic keratitis (Whitley and Roizman, 2001; Lairson et al., 2003). The virus is usually acquired during childhood and produces lifelong infections due to its ability to infect and remain latent in neurons (Kramer et al., 2003). Worldwide, nearly 60% of the population has antibodies against this virus, however only 20%–40% of those that are infected develop symptoms (Looker et al., 2015). Nevertheless, HSV-1-infected asymptomatic individuals are significant reservoirs for this virus and contribute to its transmission through shedding (Miller and Danaher, 2008; Ramchandani et al., 2016).

Regardless if the individual is symptomatic or asymptomatic after infection with HSV-1, the lifelong presence of this virus in the organism may produce in some hosts alterations in cellular processes that are required for normal neuronal cell function, which could eventually lead to pathology in the brain in a fraction of seropositive persons (Zambrano et al., 2008; Martin et al., 2014b). This notion is supported by the fact that some studies have reported the presence of HSV-1 DNA in up to 65%–75% of the brains of seropositive individuals, without clinical signs of active infection or neurological illnesses (Baringer and Pisani, 1994; Mori, 2010). The fact that HSV-1 is not invisible to the immune system and that immune cells are commonly found adjacent to infected cells, suggests scenarios in which immune cells infiltrating the CNS may somewhat contribute to chronic inflammatory processes that can be detrimental to the function of this tissue (White et al., 2012; Van Velzen et al., 2013; Ma et al., 2014). On the other hand, because the immune system of an individual tends to decay upon aging, opportunities arise for HSV-1 to reactivate in the organism and spread to tissues such as the brain. These observations have led to the notion that infection with HSV-1 may promote, or contribute to neurodegenerative disorders in humans (Dobson et al., 2003; Otth et al., 2009; Martin et al., 2011; Buscarinu et al., 2017). This idea is further reinforced by studies that suggest that other herpesviruses, such as the Epstein Barr virus (EBV) and human herpesvirus-6 (HHV-6), may be related with multiple sclerosis (MS) and Alzheimer’s disease (AD), giving herpesviruses increased attention in the last decades on their potential roles in neurological diseases (Casiraghi et al., 2012, 2015; Leibovitch et al., 2018). However, given that HSV-1 is highly prevalent in the human population and that neurodegenerative disorders are somewhat present at low frequencies in the population, a direct causal link between this virus and such type of diseases has been difficult to establish (Harris and Harris, 2015; Hogestyn et al., 2018). Nevertheless, with the advent of novel experimental techniques, high-throughput methodologies and deep sequencing approaches, host factors that could contribute to a potential relationship between HSV-1 and neurodegenerative disease could eventually be identified in the near future. This review focuses on HSV-1 infection of neurons and the brain and discusses virus modulation of cellular processes, as well as inflammation in this tissue that may favor the development of neurodegeneration in the host. Notably, HSV-1 has been associated with several neurodegenerative disorders, such as MS and AD. Here, we review this relationship and discuss recent epidemiological and pathophysiological aspects of HSV-1 and neurodegeneration (Dobson et al., 2003; Otth et al., 2009; Martin et al., 2011; Smyk et al., 2014; Buscarinu et al., 2017; Hogestyn et al., 2018).

## HSV-1 Replication and Infection of the Nervous System

### HSV-1 Replication in Epithelial Cells and Neurons

HSV-1 can alternate between a lytic infection phase that produces infectious virions, or a latent state characterized by undetectable levels of viral particles in the individual (Whitley and Roizman, 2001). Indeed, after initial infection of the epithelium in the exposed area, the virus gains access to the termini of sensory neurons that innervate the skin and reaches the cell body of these cells by retrograde transport through axons (Antinone and Smith, 2010). During facial infections that affect the mouth, face or eyes, viral progeny from HSV-1 replication in the epithelium will reach the cell bodies of sensory and autonomic nerve terminals of neurons in trigeminal ganglia (TG). While virus present in the site of infection will be cleared throughout the infection process, virus within neurons will enter a latency phase in which viral DNA remains as an episome in the nucleus of neurons with reduced-to-none virus protein expression (Nicoll et al., 2012). Remarkably, latency is characterized by the transcription of only one viral RNA transcript from the viral genome, which is non-coding and is termed the latency-associated transcript (LAT; Nicoll et al., 2016). Importantly, in latently-infected cells LAT is processed into miRNAs that silence the expression of viral genes that are required for productive virus replication (Umbach et al., 2008). Nevertheless, sporadic expression of lytic viral genes in the TG during latency in the form of mRNA has been reported by several groups (Feldman et al., 2002; Margolis et al., 2007; Ma et al., 2014), which was followed in some cases by protein synthesis suggesting that latency is likely a more dynamic process than previously thought (Du et al., 2011; Kim et al., 2012). Interestingly, LAT has been reported to be involved in neuron survival, as it displays anti-apoptotic properties, which is further discussed below (Perng et al., 2000; Henderson et al., 2002; Shen et al., 2009). Importantly, during the latent state epigenetic markers associated to the active transcription of viral genes have been identified in the LAT promoter in neurons (i.e., particular acetylation patterns at histone H3; Kubat et al., 2004). In contrast, the promoters of lytic viral genes were found to display methylations associated to heterochromatin (Cliffe et al., 2009; Cliffe and Wilson, 2017).

Noteworthy, HSV-1 latency has been observed to be concentrated at specific sites in the CNS in studies consisting of a mouse model of herpes simplex encephalitis (HSE). Mice that survived an acute phase of infection showed LAT mainly concentrated within the lateral ventricles and the hippocampus (ependymal zone), as well as the brainstem 30- and 60-days post-infection (Menendez et al., 2016). Moreover, the ependymal region in the brain evidenced HSV-1 lytic gene transcripts being expressed at these time-points post-infection, in contrast to the brainstem and TG, in which the expression of lytic genes was decreased (Menendez et al., 2016). Interestingly, this study proposes the hypothesis that a specific tropism of HSV-1 to the ependymal zone may be linked to chronic inflammatory responses in the brain and that this zone may have particular conditions that provide an environment that enhances viral persistence, potentially leading to neurodegeneration (Webb et al., 1989; Conrady et al., 2013). A more recent study showed that the ependymal zone harbors neural progenitor cells that are vulnerable to acute HSV-1 infection and viral lytic-associated proteins were detected in these cells during latency (Chucair-Elliot et al., 2014).

Importantly, the host immune response against the virus has been reported to be involved in the maintenance of a latent state by HSV-1 in neurons. Indeed, HSV-1-infected neurons have been shown to be surrounded by T cells in the TG, presumably limiting viral reactivation which would otherwise lead to lytic replication of the virus, thus hampering the generation of infectious virions from these cells (Liu et al., 1996; Verjans et al., 2007). HSV-1 specific CD8^+^ in contact with TG neurons were shown to block viral reactivation through the release of granzymes that degrade viral proteins (Khanna et al., 2003; Van Velzen et al., 2013). In contrast, viral persistence in the ependymal zone of the brain was related to T cells expressing exhaustion markers [LAG-3, TIM-3, programmed death-1 (PD1), CD160 and KLRG-1]. Furthermore, isolated T cells were unable to control HSV-1 infection *ex vivo* and secreted less interferon (IFN)-γ in comparison to T cells isolated from TG (Wherry and Kurachi, 2015; Menendez et al., 2016).

Because the immune system plays an important role in controlling HSV-1 reactivation from the brain, episodes of immune-depression such as concomitant infections or stimuli, such as fever episodes may reactivate HSV-1 from neurons and allow the virus to enter a lytic replication cycle (Sawtell and Thompson, 2016). During the lytic phase of HSV-1, either in neurons or epithelial cells the virus expresses its genes in a cascade-dependent manner, with three major waves of transcription: first, the expression of immediate early genes (IE or alpha genes), followed by the expression of early genes (E or beta genes) and lastly, late genes (L or gamma genes). Furthermore, the latter are sometimes sub-divided into late-early and late genes (or gamma-1 and gamma-2 genes, respectively; Honess and Roizman, 1974). For IE mRNAs, a viral transactivator called VP16 plays an important role in promoting their transcription by binding to cellular factors namely the octamer-binding protein 1 (Oct1) in epithelial cells and the host cell factor-1 (HCF-1), both in epithelial and neuron cells (Herrera and Triezenberg, 2004; Suazo et al., 2015). Some IE viral genes play key roles in the subversion of the host cellular antiviral response. As IE proteins are expressed, some of them will act as transcription factors for E viral genes, promoting their transcription into mRNAs that play roles in viral processes, such as DNA replication (Suazo et al., 2015). Finally, late gene expression occurs thanks to the transactivation properties of viral beta genes (Honess and Roizman, 1975). These later genes encode, among others, for structural components of the virion, such as capsid, tegument, and viral surface proteins (Honess and Roizman, 1974; Herrera and Triezenberg, 2004). Once viral proteins that compose the virion are produced, these viral elements will travel within neurons from the cell body to axonal terminals in an anterograde manner. Interestingly, two models have emerged for this process in neurons and are termed “married” and “separate” because of the mechanism of action. While in the married model HSV-1 particles travel with viral glycoproteins as a whole virion through the axons together, in the separate model HSV particles are transported in axons separated from the viral envelope and glycoproteins (Wisner et al., 2011). Nevertheless, in both cases new infectious viral particles will be released at the original site of infection, which will promote the infection of new epithelial cells within the surroundings and likely disseminate virus onto adjacent, non-infected neurons (Halford et al., 1996). This process will promote the maintenance of a continuous pool of HSV-1-infected neurons in the individual.

### HSV-1 Infection of the Central Nervous System

Herpetic simplex encephalitis (HSE) is produced by the active replication of HSV-1 in neuronal cells in the brain (Gnann and Whitley, 2017). Importantly, acute HSE induced by HSV-1 produces neuronal cell death by necrosis or apoptosis and usually relates to the temporal and frontal lobes of the brain, as well as the insular cortex of the cerebral hemispheres (Bradshaw and Venkatesan, 2016). Death of neurons, astrocytes and oligodendrocytes occurs within 7 days post-infection and histopathological examination shows areas of mononuclear inflammation in a mouse model of HSE (Armien et al., 2010). Therefore, both cytolytic viral replication and immune factors will be involved in the disease. Noteworthy, despite HSE treatment with acyclovir a high percentage of survivors will display numerous sequelae associated to neurological involvement, such as epilepsy, amnesia or cognitive and behavioral alterations (Misra et al., 2008; Riancho et al., 2013). It has been hypothesized that immune-mediated mechanisms may be key players in HSE relapses that induce neurologic damage (Valencia et al., 2004; Prüss, 2017).

**Figure 1:**
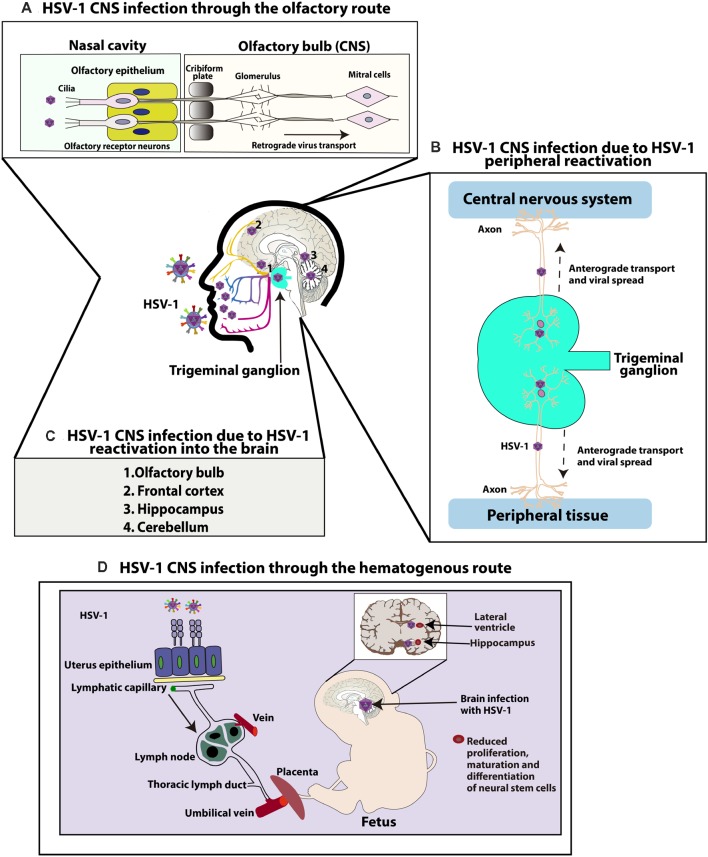
Central nervous system (CNS) infection with herpes simplex virus type 1 (HSV-1).** (A)** HSV-1 CNS infection through the olfactory route: HSV-1 can infect the termini of olfactory neurons enervating the nasal epithelium and access the CNS by retrograde axonal transport through neurons until reaching the olfactory bulb in the brain. **(B)** HSV-1 can also infect the CNS because of HSV-1 peripheral reactivation. HSV-1 can reactivate from neurons in the trigeminal ganglia (TG) and reach either the skin or CNS through anterograde transport. **(C)** HSV-1 can also reach different regions of the CNS because of HSV-1 reactivation within the brain. Reactivation of latent virus within the CNS has been reported to reach the cerebellum, olfactory bulb, frontal cortex, or hippocampus. **(D)** Finally, HSV-1 can infect the CNS through a hematogenous route. HSV-1 can infect the CNS of fetuses because of HSV-1 infection in the mother, through the bloodstream by accessing the placenta. Once the fetus is infected, HSV-1 accesses the brain and has been reported to infect the hippocampus. Neural stem cells (NSCs) in the subventricular zone of the lateral ventricle and subgranular zone of the hippocampus have been shown to be infected by HSV-1, affecting the maturation and proliferation, as well as differentiation of these cells.

While nearly 30% of HSE cases are related to primary HSV-1 infection (commonly observed in children and adolescents), 70% of cases of HSE are attributed to a previous HSV-1 infection and viral reactivation (mostly observed in adults; Steiner and Benninger, 2013). Regarding how HSV-1 reaches the CNS to develop HSE, several routes and mechanisms have been proposed both, as a consequence of primary infection or due to viral reactivations (Figure 1; Bradshaw and Venkatesan, 2016). For primary infections, both the olfactory and hematogenous routes have been proposed (Burgos et al., 2005; Jennische et al., 2015). Indeed, for neonatal HSV-1 infections the olfactory route is frequently deemed responsible and widely described as the result of close contact between the newborn olfactory tissue and HSV-1 virions present in the birth canal of the mother at the time of birth (Burgos et al., 2006). Consistent with this notion, animal models have shown spread of HSV-1 from the nasal cavity to the CNS after infection of the olfactory epithelium, which is connected with the olfactory bulb and consequently the limbic system, resulting in focal encephalitis in the brain (Figure 1A; Twomey et al., 1979; Dinn, 1980). However, another study in mice suggests that vertical transmission is predominantly hematogenous (Figure 1D; Burgos et al., 2006). This study showed that offspring born to HSV-1-infected mothers harbored HSV-1 proteins and DNA, mainly in the hippocampus in the CNS. Moreover, the placenta also showed high number of viral genomes, indicating that HSV-1 can reach the brain of fetuses by this route through the maternal bloodstream. Finally, the authors reported that the administration of acyclovir in HSV-1-infected mothers reduced vertical transmission of this virus (Burgos et al., 2006).

Another route by which HSV-1 may gain access to the brain is through peripheral viral reactivations followed by subsequent anterograde axonal transport (Figure 1B; Kramer and Enquist, 2013). A study with patients with HSE simultaneously compared HSV-1 isolates from the mouth and brain of infected individuals and showed that five out of eight isolate pairs were identical, as determined by restriction endonuclease analyses indicating that HSV-1 infection in a latent state in the TG acquired in a previous orolabial infection may reactivate from this site and reach neurons in the CNS (Whitley et al., 1982).

Finally, reactivation of latent virus from the CNS may also seed infection to other sites within the brain (Figure 1C; Stroop, 1986). Post-mortem studies have reported the presence of HSV-1 genomes in brain tissues of individuals without any known neurologic disease, suggesting the possibility that HSV-1 could establish latency in the CNS (Olsson et al., 2016). Although sensory ganglia are understood to be the primary source of virus establishing latency, recent studies using a modified *ex vivo* tissue explant reactivation assay have found that 80% of brainstem explants display viral reactivation following latent infection with KOS or McKrae HSV-1 viruses, indicating that the CNS can also be an effective source of infectious HSV-1 from which the virus reactivates (Chen et al., 2006). Moreover, hyperthermia-induced viral reactivation *in vivo* has also been evidenced in the brainstem before its detection in the TG, with the virus reactivating in this study more frequently from the brainstem than the TG (Yao et al., 2014). Latent viral genomes were also detected in the cerebellum, olfactory bulbs, frontal cortex, and hippocampus of these mice (Yao et al., 2014). According to this information, it is possible that latent HSV-1 in the brain may be a source of productive reactivations in this tissue, which could cause HSE in some susceptible individuals.

## Proposed Factors for an Association Between HSV-1 Infection and Neurodegeneration

### HSV-1 Modulates Apoptosis-Related Pathways

Apoptosis is a cellular death program activated in response to external or internal cellular stimuli that lead to controlled autodestruction of the cell (Webb et al., 1997). Some macroscopic key features of this process are nuclear fragmentation, chromatin condensation and the presence of apoptotic bodies in the cell cytoplasm (Assunção Guimarães and Linden, 2004). Overall, apoptosis can be triggered by extrinsic or intrinsic signaling pathways and executed by a family of cysteine proteases known as caspases (Thornberry and Lazebnik, 1998).

The extrinsic pathway is known to respond to external stimuli and is triggered by the binding of ligands to membrane death receptors, mainly belonging to the tumor necrosis factor receptor (TNFR) superfamily that overall lead to the activation of caspases-8 or -3 (Bodmer et al., 2002; Lavrik et al., 2005; Pennarun et al., 2010). This results in either cell death or the stimulation of the mitogen-activated protein kinase/c-Jun N-terminal kinase (MAPK/JNK) pathway that can alternatively lead to cell survival, proliferation or ultimately induce apoptosis by enhancing the activity of pro-apoptotic signaling molecules in response to prolonged cellular damage (Lu and Xu, 2006; Declercq et al., 2009). Importantly, nuclear factor-κB (NF-κB) is also activated by the death-inducing signaling complex (DISC) and can trigger apoptosis or the expression of anti-apoptotic genes that are members of the cellular FLICE-inhibitory protein (cFLIP) family, BCL-2 family or cIAP family (Saleem et al., 2013). Interestingly, caspase activation by the extrinsic pathway has been reported to be able to stimulate the intrinsic pathway of apoptosis, due to the cleavage of the pro-apoptotic protein Bid into a truncated form termed tBid (Schulze-osthoff et al., 1998).

On the other hand, the intrinsic apoptosis pathway mainly consists on the stimulation of apoptosis by intracellular stimuli that may occur by irreparable cellular damage or cells being hijacked by pathogens (Lamkanfi and Dixit, 2010). This signaling pathway leads to the permeabilization of the outer membrane of mitochondria with the concomitant release of cytochrome c to the cytoplasm (Kroemer et al., 2007). Cytochrome c in turn binds to Apaf-1 and triggers the assembly of the apoptosome (Yuan and Akey, 2013), which interacts with pro-caspase-9 to produce its cleavage into caspase-9 that activates a caspase cascade leading to apoptosis by caspases-3/7 (Friedman and Nunnari, 2014). This pathway also involves a crosstalk between the endoplasmic reticulum (ER) and mitochondria. Under ER stress, an unfolded protein response (UPR) is activated, which elicits a cascade of signaling events that induce apoptosis (Shore et al., 2011). Indeed, ER stress induced by several pathophysiological stimuli can lead to JNK activation through Ca^2+^ release (Verma and Datta, 2012). JNK activation produces further release of Ca^2+^, which generates an active redistribution of the Bax/Bak pro-apoptotic proteins in the mitochondria (Lei and Davis, 2003). In turn, Bax/Bak proteins allow the release of the apoptotic proteins cytochrome c and AIF proteins (Schulz et al., 1997). Alterations in membrane potential of mitochondria, given by these processes have also been reported to lead to the formation of a pore in this organelle with the consequent activation of caspase- and -3, both activated by cytochrome c release (Kroemer et al., 2007). Importantly, JNK can also activate the pro-apoptotic protein Bim and block anti-apoptotic Bcl-2 proteins (Bcl-2, Bcl-xl, Mcl-1, A1; Verma and Datta, 2012). Ultimately, the balance between pro- and anti-apoptotic proteins will control the susceptibility of a cell to undergo apoptosis. Importantly, neuronal survival is continuously stimulated by trophic factors that function through specific receptors that are regulated by MEK/ERK and that lead to c-AMP response element binding protein (CREB) activation, a key regulator of several cellular processes (Wang et al., 2003). However, apoptosis can sometimes result by the interference of the strict regulation of these pathways (MEK/ERK/CREB) when danger or damage stimuli are present (Wang et al., 2003).

Noteworthy, although apoptosis is considered to be a result of neurodegeneration, alterations in signaling pathways related to apoptosis have been widely described to be implicated in neurodegenerative diseases, such as AD (Obulesu and Lakshmi, 2014), Parkinson’s disease (Lev et al., 2003), and amyotrophic lateral sclerosis (Sathasivam et al., 2001). Hence, HSV-1 modulation of neuronal apoptosis both, during acute and latent infection could eventually relate to alterations of neuronal processes that lead to neuron damage and brain disease (Guégan and Przedborski, 2003; Hickey and Chesselet, 2003; Nguyen and Blaho, 2007; Gelders et al., 2018).

During acute brain infection in HSE, HSV-1 is known to induce apoptosis in neural cells that contribute to virus-induced CNS pathogenesis (Debiasi et al., 2002). In most of the brains with HSE analyzed, neurons showed an apoptotic state, which was characterized by positive terminal deoxynucleotidyl transferase-mediated dUTP nick-end labeling (TUNEL; Debiasi et al., 2002). The mechanism by which HSV-1 induces apoptosis in rat hippocampal neuron cultures has been shown to be through the activation of JNK-related pathways (Perkins et al., 2003). Indeed, hippocampal neurons displayed significant DNA fragmentation (TUNEL^+^), and activated caspase-3 when determined by immunohistochemistry (Perkins et al., 2003). A role for JNK in the activation of apoptosis observed in these cells was supported thanks to the use of the JNK inhibitor SP600125, which was able to abolish apoptosis in neurons infected with HSV-1 (Perkins et al., 2003). Importantly, similar results were obtained with brain tissues obtained from patients with HSE (Perkins et al., 2003). In addition, HSV-1 has been reported to induce apoptosis in brainstems of mice infected with this virus, in which significant TUNEL-staining was observed at day 6 post-infection, although with low levels of detectable infectious virus, suggesting death of cells that were bystander to those infected with HSV-1 (Shaw et al., 2002). Furthermore, the viral protein infection cell protein 0 (ICP0), which is an immediate HSV-1 early protein has been shown to act as an activator of apoptosis during HSV-infection. Expression of ICP0 alone was shown to be necessary and sufficient to trigger cell death-associated signaling cascades in HEp2 (human, epithelial cervix) and Vero cells (green monkey, kidney epithelial), which was evidenced with a mutant virus devoid of ICP0 unable to induce apoptosis in infected cells (Sanfilippo and Blaho, 2006).

**Figure 2:**
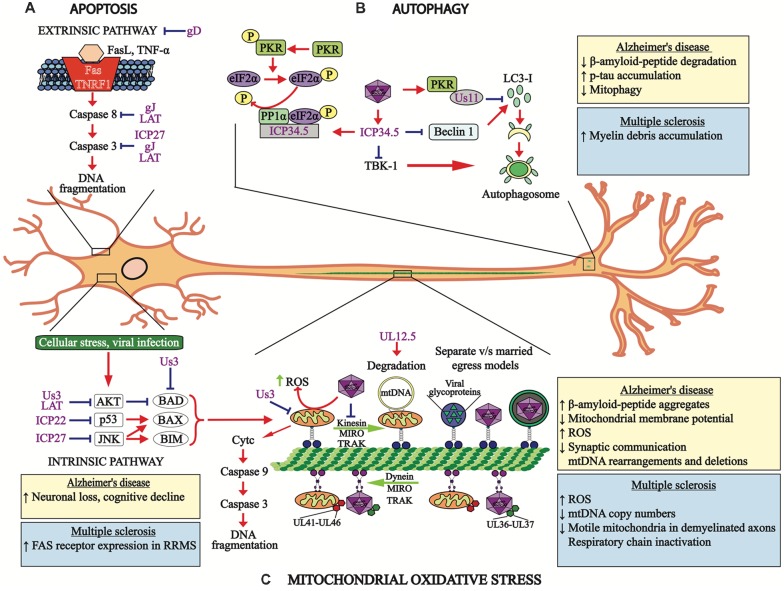
HSV-1 modulates cellular processes. **(A)** HSV-1 modulates apoptosis-related pathways. The apoptosis extrinsic (upper section) and intrinsic (lower section) pathways are modulated by HSV-1 proteins, such as the immediate early proteins infection cell protein 22 (ICP22), ICP27 and US3, late viral proteins glycoprotein D (gD) and gJ, as well as the latency-associated transcript (LAT) transcript, which hamper events leading to apoptosis at different stages of signaling cascades and at distinct time-points after infection. **(B)** HSV-1 modulates autophagy. Viral protein US11 interaction with PKR inhibits LC3-I conversion into LC3-II. HSV-1 ICP34.5 inhibits autophagosome formation by blocking beclin-1 and subsequent LC3-I conversion into LC3-II, which is necessary for proper autophagosome function. The viral protein ICP34.5 also inhibits TBK-1, which blocks autophagosome formation. **(C)** Mitochondria oxidative stress is also modulated by HSV-1 infection. Both, mitochondria and HSV-1 transport are mediated by retrograde and anterograde processes involving microtubules. Importantly, HSV-1 infection blocks the transport of mitochondria and viral tegument proteins (UL41 and UL46) migrate with this organelle within infected neurons. Retrograde transport of the HSV-1 capsid is accompanied by the viral tegument proteins UL36 and UL37. Anterograde transport of HSV-1 components to neuron termini can be mediated either separately or in a conjoint manner, which are known as the “married” and the “separate” models. The viral protein UL12.5 produces mitochondria DNA degradation and the viral protein US3 protein blocks the electron transport chain within this organelle. The boxes show the cellular processes or pathologies that occur in Alzheimer’s disease (AD) (yellow boxes) or multiple sclerosis (MS; blue boxes) associated with apoptosis, autophagy and mitochondria oxidative stress. RRMS, relapsing-remitting MS.

On the other hand, the frequent finding of HSV-1 DNA in the brains of individuals that do not display neurological diseases, suggests that CNS infection with this virus does not necessarily translate into cell death (Jamieson et al., 1991). Interestingly, because apoptosis is used by the host as an antiviral defense strategy in order to eliminate virus, HSV-1 encodes numerous viral determinants for evading it (Figure 2A), which overall may favor virus persistence in the host by remaining in a viable substrate as a long-term niche for latency, which could produce chronic inflammation due to recurrent asymptomatic reactivations (Perng et al., 2000). Importantly, both protein and RNA-based viral-determinants have been reported to have anti-apoptotic effects in HSV-1-infected cells. For instance, the gene products of glycoproteins J and D (gJ, gD) have been reported to modulate cellular apoptosis during HSV-1 infection of the SK-N-SH human neuron cell line (neuroblastoma), as mutant viruses lacking any of the genes encoding for these proteins led to cell apoptosis early after infection, which was abolished when gD and gJ were complemented in *trans* (Zhou et al., 2000). Furthermore, HSV-1 lacking the gJ gene *Us5* was hampered at inhibiting caspase-3/8 activation after Fas ligation or UV irradiation (Jerome et al., 2001). Although the *Us6* viral gene, which encodes the gD has also been reported to block apoptosis in these cells, the molecular mechanism involved in inhibiting apoptosis by this viral glycoprotein has not been completely elucidated, although an association with NF-κB activation and increased expression of NF-κB-dependent anti-apoptotic genes, such as c-IAP2, FLIP and survivin has been suggested as a mode of action in U937 monocytoid cells (Medici et al., 2003; Marino-Merlo et al., 2016). U937 cells infected with wild-type or UV-inactivated HSV-1 displayed inhibition of Fas-induced apoptosis, suggesting that a structural viral component may be exerting this effect (Marino-Merlo et al., 2016). Moreover, when U937 cells were co-cultured with gD-expressing transfected cells, before anti-Fas addition a significant inhibition of Fas-mediated apoptosis was observed, which was abolished by transfection of a dominant negative inhibitor of NF-κB activity (IκBα), thus supporting a key anti-apoptotic role for gD (Medici et al., 2003). Furthermore, more recently glycoprotein E (gE) has also been reported to act as an inhibitor of apoptosis in epithelial cells, which was achieved by triggering ERK1/2 activation and was associated with the degradation of the pro-apoptotic protein Bim (Figure 2A; Pontes et al., 2016).

Another HSV-1 protein involved in negatively modulating apoptosis is ICP22 (Nguyen et al., 2005). A virus with ICP22 deleted was shown to induce more apoptosis than the wild-type virus (Nguyen and Blaho, 2007). ICP22-mediated inhibition of apoptosis likely involves p53, a cellular transcription factor that controls apoptosis by activating Bax or inhibiting Bcl-2, and that has been shown to be antagonized by ICP22 which promotes cell survival (Figure 2A; Pietsch et al., 2008; Maruzuru et al., 2013).

Likewise, the viral ICP27, an IE protein has been reported to block caspase 3-associated apoptosis, as evidenced with a mutant of HSV-1 that has ICP27 deleted (Aubert and Blaho, 1999). Inhibition of apoptosis by ICP27 was shown to require an amino acid sequence close to the N-terminus of the protein, which activates p38 and JNK signaling in CV-1 cells (Hargett et al., 2005). Additionally, in this same study a mutant virus with other IE proteins deleted were assessed in order to determine if ICP27 alone was sufficient to activate p38 and JNK. The results of this study showed that viruses lacking other IE viral proteins (ICP22 or ICP47) activated p38 and JNK similar to the wild-type virus, suggesting that these proteins are not necessary for p38 and JNK activation (Hargett et al., 2005). Importantly, JNK activation is known to stimulate NF-κB signaling that is associated with inhibition of apoptosis (Figure 2A; Hargett et al., 2006). In contrast, ICP27 has also been reported to prevent the phosphorylation and degradation of IκBα, the endogenous inhibitor of NF-κB, which would then block apoptosis (Kim et al., 2008).

On the other hand, miRNAs derived from the processing of the LAT have also been reported to modulate apoptosis in infected cells, notably in neurons where the virus establishes latency and expresses this transcript during this phase. LAT expressed in a plasmid was shown to inhibit caspase-8 and caspase-9-induced apoptosis (Figure 2A; Henderson et al., 2002), which lead to inhibition of CD8^+^ T cell-killing of latently infected neurons, as LAT expression blocked granzyme B-induced cleavage of caspase 3, thus protecting C1300 and Neuro2A cells (Jiang et al., 2011). Moreover, two small RNAs (sRNAS) encoded by LAT were recently shown to prevent cold shock-induced apoptosis in mouse neuroblastoma cells by a mechanism that remains to be determined (Shen et al., 2009). Finally, HSV-1 LAT can also up-regulate the levels of protein kinase B (AKT), a protein that promotes cell survival by inactivating the pro-apoptotic proteins Bad, Bax and caspase-9 (Cooray, 2004). Through AKT, LAT negatively regulated caspase-3 activation and increases the ratio of Bcl-2/tBid in neurons enhancing cell survival (Figure 2A; Carpenter et al., 2015). Thus, inhibition of apoptosis may play a key role in neurodegeneration by HSV-1 favoring the establishment of latency and persistence with later reactivations and spread into the neuronal tissue, leading to neuron damage in a long-term manner.

On the other hand, the AMPK/Sirt1 axis has been shown to be modulated during HSV-1 neuronal infection and to interfere with apoptosis signaling (Martin et al., 2014b). Two hours post infection, p-AMPK levels were declined and Sirt1 protein remained non-induced in contrast to p53 levels, which increased demonstrating a pro-apoptotic state in neurons (Martin et al., 2014b). However, 4–8 h post infection, HSV-1 positively regulated AMPK/Sirt1 axis showing an increase in Sirt1 activity and a reduction in the levels of acetylated p53 thus, promoting an anti-apoptotic state (Martin et al., 2014b). These results suggest that HSV-1 can modulate this pathway at different time-points after infection by interfering with apoptotic signaling events to favor its replication at early times in the replication cycle and after supporting neuronal survival for its persistence in a latent state.

Taken together, HSV-1 induces and inhibits apoptosis-related pathways at multiple steps after neuron infection (Aubert and Blaho, 2001). This modulation of apoptosis could contribute to the capacity of this virus to manipulate neuronal survival, as well as functions related either directly or indirectly with neurodegenerative processes. Indeed, HSV-1 is able to modulate several host processes and take advantage of numerous signaling pathways in order to favor its persistence and shedding throughout the CNS (Kramer et al., 2003; Villalba et al., 2012; White et al., 2012). Deepening on the knowledge of these processes could allow the identification of new targets for pharmacological intervention of important signaling pathways involved in neurodegeneration and the treatment of neurodegenerative diseases (Leyton et al., 2015).

### HSV-1 Disrupts Autophagy-Related Processes

Macroautophagy (autophagy) is a process that involves the development of autophagosomes, double membrane-bound structures, that ultimately fuse with lysosomes to degrade cytosolic contents (Awan and Deng, 2014). It is important to point out that autophagy is involved in cellular homeostasis by removing old or damaged organelles, provide nutrients to the cell in starvation responses and also to eliminate aggregated proteins caused by misfolding disorders, which can elicit cells stress (Klionsky et al., 2007).

Importantly, autophagy dysfunction has been associated to pathogenesis in various neurodegenerative disorders. The role of autophagic pathways over the prevention of neurodegeneration has been evidenced by generating selective neural animal models, such as one in which cell-specific Atg5 deletion is controlled, as Atg5 is essential for autophagosome formation and thus, mice deficient for this protein develop progressive deficits in motor function that are accompanied with the accumulation of cytoplasmic inclusion bodies in neurons (Hara et al., 2006). The Atg7 protein is also essential for autophagy and mice that are knockout for this gene in the CNS show symptoms and signs that are similar to those evidenced during neurodegenerative disorders in humans, with significant neuronal loss in the cerebral and cerebellar cortices (Komatsu et al., 2006). Additional studies indicate that autophagy has an important role in the survival of neurons, by preventing the accumulation of irregular proteins and avoiding neurodegeneration (Hara et al., 2006; Komatsu et al., 2006). In this context, autophagy has been reported as a key regulator of neurogenesis by sustaining new neuron pools from neural stem cells (NSCs). A recent study reported high autophagy protein expression (AMBRA 1 and Beclin 1) in neural tissue enriched in NSCs, such as the sub-ventricular zone of the lateral ventricle and the subgranular zone of the dentate gyrus in the hippocampus (Yazdankhah et al., 2014; Casares-Crespo et al., 2018). Moreover, inhibition of FIP200, another autophagy-related protein, resulted in a progressive loss of NSCs *in vivo* and a deficiency in neuronal differentiation (Wang et al., 2013). Likewise, Beclin-1 heterozygosis *in vivo* resulted in cell proliferation and maturation disorders (Miller et al., 2014).

Importantly, autophagy acts as a defense mechanism against several infections promoting lysosomal degradation of the pathogen. During HSV-1 infections, shutting off cellular protein synthesis is carried out as an antiviral defense mechanism, which is achieved through the phosphorylation of the eukaryotic initiation factor 2α (eIF2α) by the RNA-activated protein kinase (PKR), therefore avoiding viral replication (Chang et al., 2002; Tallóczy et al., 2002). Moreover, eIF2α phosphorylation promotes the induction of autophagy, which is key for controlling HSV-1 infection in neurons (O’Connell and Liang, 2016), in contrast to epithelial cells, where an IFN response is sufficient to control the infection and autophagy is not required (Yordy et al., 2012). However, while autophagy protects the adult brain from viral encephalitis, contrasting results have been reported in newborn mice, where autophagy seems be harmful for the host and to promote neuronal apoptosis. These results suggest an age-dependent role for autophagy during brain infection (Wilcox et al., 2015). On the other hand, autophagy can be modulated by viruses to improve the production of viral particles during the lytic phase, or increase their persistence during a latent phase (O’Connell and Liang, 2016; Lussignol and Esclatine, 2017). In agreement with this notion, key roles for autophagy during viral latency and reactivation have been reported for infections with gammaherpesviruses (Silva and Jung, 2013). It is known that viral persistence of EBV can be stimulated by autophagy, due to its involvement in the regulation of cell survival (Pujals et al., 2015). In contrast, MHV68 reactivation was stimulated by autophagy due to its regulation in systemic inflammation, while HHV8 blocked autophagy during latency (Leidal et al., 2012; Park et al., 2016), However, studies elucidating specific roles for autophagy in the context of HSV-1 latency and reactivation are lacking.

Regarding lytic infection with HSV-1, this virus has evolved mechanisms to inhibit autophagy through its neurovirulence factor named infected cell protein 34.5 (ICP34.5, or gamma-34.5), as well as viral protein US11 (Figure 2B; O’Connell and Liang, 2016). Interestingly, viral ICP34.5 can block autophagy by several pathways, one of them is the inhibition of BECN1-mediated autophagy. Previous reports using co-immunoprecipitation assays showed that N-terminal domain of ICP34.5 binds directly to Beclin-1 autophagy protein and interferes with autophagosome biogenesis (Orvedahl et al., 2007). On the other hand, the C-terminal domain recruits the host phosphatase PP1α, which reverts eIF2α phosphorylation mediated by PKR inhibiting autophagy (Wilcox and Longnecker, 2016). More recently, another target for ICP34.5 has been reported, namely tank binding kinase 1 (TBK1; Verpooten et al., 2009). Because TBK1 is an essential autophagy-related protein which phosphorylate autophagy receptors (i.e., autophagic adaptor optineurin) to regulate the recruitment of cargo into autophagosomes (Weidberg and Elazar, 2011), it is possible that ICP34.5 could also indirectly inhibit autophagy by modulating TBK1 signaling. However, further studies are needed to confirm this hypothesis. Furthermore, US11 a tegument viral protein that is expressed late in the replication cycle of HSV-1, has been reported to interact directly with PKR and inhibit subsequent eIF2α phosphorylation (Figure 2B; Lussignol et al., 2013). A study using HeLa cells (human epithelial cervix) and fibroblast cells showed that US11 can inhibit autophagy and autophagosome formation. Moreover, the authors reported that earlier expression of US11 in cells infected with a mutant virus deleted in the ICP34.5 gene allowed the cells to inhibit virus-induced autophagy (Lussignol et al., 2013). The role of this protein in the context of neuronal infection remains to be elucidated.

Notably, HSV-1 produces accumulation of intracellular autophagosomes in human neuroblastoma cells and increases amyloid beta (Aβ) accumulation in autophagy compartments in these cells (Santana et al., 2012). Interestingly, these observations suggest a role for HSV-1 in the development of AD. Moreover, because autophagy is essential for neuron homeostasis, HSV-1 could contribute to CNS damage through the modulation of autophagy as described below, enhancing neurodegeneration.

On the other hand, a decrease in autophagy has been reported in several neurodegenerative disorders, which proposes that this process is a factor that contributes to protein accumulation and cellular toxicity due to problems in protein folding, as in AD (Menzies et al., 2011). In addition, alterations in the degradation of myelin debris could be affecting MS by promoting its extracellular accumulation (Neumann et al., 2009; Liang and Le, 2015). Also, dysfunctional autophagy could play a role in HSV-1 persistence and reactivation in neurons, similar to other herpesviruses (Silva and Jung, 2013). Hence, further studies evaluating the role of autophagy under these conditions are needed. Importantly, it has been proposed that the up-regulation of autophagy could work as a therapeutic target to treat these diseases (Thellung et al., 2018), and autophagy stimulation has been shown to significantly suppress HSV-1 infection in various cell types, as evidenced by assessing HSV-1 genomes and virus titers that indicated that inducing autophagy strongly suppresses HSV-1 infection (Yakoub and Shukla, 2015).

### HSV-1 Induces Mitochondrial Dysfunction

Mitochondria are essential organelles for energy production and also play key roles in modulating cell fate (Friedman and Nunnari, 2014). Numerous mitochondria proteins encode for components related to the respiratory chain that give rise to high amounts of energy in the cell, such as the cytochrome c oxidase subunit 1 (CO1; Nicholls and Gustafsson, 2018). Yet, mitochondria function is also essential for neuronal processes involving Ca^2+^ fluxes and homeostasis, which play important roles on the stability of action potentials in the plasmatic membrane of these organelles, promotion of protein folding through chaperones and axonal transport and synapse processes, among others (Knott et al., 2008). Importantly, mitochondria occupy about 40% of the cytoplasmic volume in neuronal cells (Fieni et al., 2012). Therefore, mitochondria imbalance has been associated with multiple neurodegenerative diseases, such as AD, Parkinson and MS, among others (Area-gomez et al., 2018; Grünewald et al., 2018; Kozin et al., 2018).

In AD, accumulation of Aβ-protein aggregates in the brain resulted in mitochondria dysfunction, which was associated with a decline in mitochondrial membrane potential and an increase in the production of reactive oxygen species (ROS; Oka et al., 2016; Rönnbäck et al., 2016). Also, in an animal model of AD, intracellular accumulation of the tau protein was shown to produce a decrease in mitophagy and subsequent mitochondria dysfunction that affected synaptic communication (Hu et al., 2016; Pérez et al., 2018). Additionally, deletion of mitochondria DNA (mtDNA) and mtDNA rearrangements were found in post-mortem human brain samples in AD (Chen Y. et al., 2016).

On the other hand, mitochondria abnormalities have been reported during the development and progression of MS disease (Kalman et al., 2007; Mao and Reddy, 2010; Patergnani et al., 2017), such as decreases in mtDNA copy numbers in the brains of patients with MS (Blokhin et al., 2008; Campbell et al., 2011). Also, anomalous mitochondria proteins, as well increased amounts of free radicals and oxidative damage were observed in MS (Mao and Reddy, 2010). In an experimental autoimmune encephalitis (EAE) model, mitochondria dysfunction was induced by protein inactivation of complexes of the respiratory chain (Qi et al., 2006; Mahad et al., 2008). Interestingly, mitochondria content within axons oscillated between myelinated, remyelinated and demyelinated axons in post-mortem tissues from patients with MS, with mitochondria content increased in remyelinated axons (Zambonin et al., 2011). Also, the number of mobile mitochondria did not differ between remyelinated and myelinated axons, but their numbers were significantly less in demyelinated axons (Zambonin et al., 2011). Importantly, it has been reported that cell infection with HSV induces mitochondrial dysfunction (Figure 2C; Ohta and Nishiyama, 2011; Martin et al., 2014b), such as morphological changes and altered migration patterns in epithelial cells, where mitochondria migrate toward the perinuclear region of cells to join UL41 and UL46 tegument viral proteins, possibly to favor its replication cycle (Murata et al., 2000).

HSV-1 has also been reported to alter mitochondria respiration in infected neurons, as evidenced through a block in the mitochondria electron-transport chain between complexes II and III at 12 h post-infection. Importantly, this process was mediated by the US3 viral protein (Derakhshan et al., 2006). Furthermore, a decrease in ATP and lactate levels in infected cells, as well as lower mitochondria membrane potential was observed at a later time post-infection (24 hpi; Derakhshan et al., 2006). Additional reports have suggested that this process would be associated with an induction of the degradation of host mtDNA after HSV-1 infection and that this process would be mediated by the viral protein UL12.5 (Figure 2C; Saffran et al., 2007; Corcoran et al., 2009).

Interestingly, although induction of mitochondria biogenesis has been reported in HSV-1-infected neurons at 18 hpi, as determined by an increase in molecular markers, such as PGC1α and TFAM (Martin et al., 2014b), mitochondria function was impaired upon HSV-1 infection in neuronal cells, in which the motility and morphology of mitochondria were severely affected through a mechanism that involved increased intracellular Ca^2+^ and reduced recruitment of kinesin-1, a protein necessary for mitochondria transport along microtubules (Kramer and Enquist, 2012). More recently, a genome-wide transcriptomic study in post-mortem human HSE brain tissues showed a greater reduction in mitochondria transcripts in HSE brain tissues than control tissues (Wnęk et al., 2016). Similar results were evidenced in *in vitro* cultures with astrocytes infected with HSV-1, in which astrocytes displayed mitochondria damage with ultra-structural changes and reduced CO1 transcripts (Wnęk et al., 2016). These reports suggest that interference with mitochondria function by HSV-1 could significantly contribute to the pathogenesis of neurodegenerative disorders.

### HSV-1 Induces Oxidative Stress

Multiple studies have reported that infection with HSV-1 increases the levels of ROS, a marker of oxidative damage in the cell, in brains infected with this virus, which could contribute to neurotoxicity associated with HSE (Kavouras et al., 2007; Schachtele et al., 2010). A recent study reported that ROS levels in microglia are increased after infection with HSV-1 and that this process is dependent on Toll-like receptor 2 (TLR2) and p38 MAP kinase, as well as ERK1/2 signaling pathways (Schachtele et al., 2010). Furthermore, another study assessing HSV-1-infected neural cells reported an increase in ROS levels after infection with this virus (Kavouras et al., 2007).

Other markers related to oxidative stress are lipid peroxidation products, such as 4-hydroxy-2-nonenal (HNE) which is released at high levels early after the infection of neurons with HSV-1. HNE is one of the main products produced upon lipid peroxidation, which was also reported in HSV-1 latent infection (Valyi-Nagy et al., 2000; Kavouras et al., 2007). Moreover, quantification of the levels of F4-neuroprostanes (F4-NP) and F2-isoprostanes (F2-IP), which are products derived from arachidonic acid (AA) and docosa-hexaenoic acid (DHA), has been suggested to provide information on the magnitude of oxidative damage occurring in the brain (Patel et al., 2001). In this regard, murine brains undergoing HSV-1 encephalitis displayed chronic inflammation accompanied by moderately-elevated levels of F2-IP, while F4-NP levels remained normal (Milatovic et al., 2002). Furthermore, two other markers associated with oxidative damage detected during HSE in mice were inducible nitric oxide synthase (iNOS) and the enzyme heme oxygenase-1 (HO-1; Marques et al., 2008a). Indeed, HSV infection increased the expression of these genes in the brain of mice 7 days post-infection, which remained elevated for 21 days. Although early up-regulation of these enzymes are involved in the host antiviral response, an overproduction of nitric oxide could be detrimental to the CNS and lead to brain damage (Barañano and Snyder, 2001). In addition, 3-nitrotyrosine, 8-hydroxydeoxyguanosine (8-OH-dG) and 8-isoprostane levels were also found to be elevated in the brain tissue of mice upon HSV-1 infection and microglia presented increased levels of iNOS expression (Marques et al., 2008a).

Notably, recent studies indicate that oxidative stress is associated with neurodegenerative diseases, such as AD and MS (Di Domenico et al., 2017; Umeno et al., 2017; Feitosa et al., 2018). For instance, such studies show that AD patients overall display increased ROS levels, while a reduced antioxidant capacity (Gubandru et al., 2013; Yang et al., 2016; Wojsiat et al., 2018). Importantly, ROS generation is associated with Aβ-protein aggregates, which are known to promote synaptic dysfunction (Ahmad et al., 2017; Hilt et al., 2018). Furthermore, the host HO-1 enzyme was found to be elevated in microglia in an AD model (Xing et al., 2014). Noteworthy, pharmacological induction of HO-1 has been reported to elicit neuroprotective effects against the neurotoxic Aβ-protein aggregates (Wang et al., 2016) and hamper the replication of HSV in a human neuronal cell line, although this was determined using HSV-2 (Ibáñez et al., 2017). Also, NO derived from the dimerization of neuronal NOS (nNOS), a component that has been reported to have neuroprotective effects, was found to be altered in AD and likely contribute to the disease (Kwon et al., 2016). Additionally, iNOS has been shown to be upregulated in AD, resulting in NO production and an increase in 3-nitrotyrosine levels, a process that has been found to be mediated by Aβ-protein (Kummer et al., 2011; Di Domenico et al., 2012). Moreover, lipid peroxidation is increased in AD, as high levels of F-neuroprostanes, F-isoprostanes and 4-HNE were detected in such patients, all products associated with Aβ-peptide (Völkel et al., 2006; Reed et al., 2008; Montine et al., 2011; Gwon et al., 2012; Miller et al., 2014). In MS elevated levels of ROS production have also been reported, which contribute to demyelination and axonal damage (Choi et al., 2015). Interestingly, in this case HO-1 expression was found to be downregulated in peripheral blood mononuclear cells (PBMCs) of MS patients (Fagone et al., 2013) and lipid peroxidation was associated with MS (Mattsson et al., 2007). Noteworthy, high levels of F-NP and F2-IP, have also been detected in MS patients (Mattsson et al., 2007; Miller et al., 2014). However, the roles of oxidative stress in neurodegenerative disorders remain controversial due to the fact that antioxidant therapies have not been found to improve these disease (Mazzanti and Di Giacomo, 2016).

### HSV-1 Alters the DNA Damage Response

The DNA damage response (DDR) is an essential pathway of the cell that is responsible for maintaining genome stability (Hakem, 2008). When DNA lesions occur, such as double strand breaks (DSBs) or single strand breaks (SSBs), this system produces a cell-cycle arrest and activates DNA repair networks (Giglia-Mari et al., 2011). Importantly, non-perfect repair of DSBs or SSBs, such as non-homologous end joining (NHEJ) can produce DNA mutations that may ultimately lead to cellular death by apoptosis, although they may also contribute to cell survival (Hakem, 2008; Giglia-Mari et al., 2011).

Importantly, impaired DNA repair has been widely documented in several neurodegenerative disorders (Madabhushi et al., 2014). For instance, NHEJ has been reported to be deficient in AD neurons (Kanungo, 2016). Additionally, transgenic mice used as a model of AD disease were shown to have reduced BRCA1 levels, an important DNA repair factor, which was accompanied by an increase in DSB and synaptic impairments, among other symptoms associated to AD (Suberbielle et al., 2015). Moreover, it has been suggested that polymorphisms in nucleotide excision repair genes may be associated with a risk of undergoing MS (Briggs et al., 2010), and another study found a positive correlation between the levels of phosphorylated γH2AX (a marker for DNA damage) with MS severity (Grecchi et al., 2012).

Some viruses have been reported to modulate different DDR pathways resulting in both, up- or down-regulation of several factors related to this pathway that favor their replication cycles (Turnell and Grand, 2012). In this regard, a study reported that DDR is beneficial for HSV-1 viral replication in non-differentiated cells, but was abolished in neuronal cells (Lilley et al., 2005). However, contradictory results have been recently reported, in which HSV-1 infection produced SSBs and DSBs in rat embryo cortical neurons and reduced the expression of Ku80, a component involved in NHEJ which is involved in DSB repair (De Chiara et al., 2016). It is possible that DDR dysfunction in neurons during HSV-1 infection may contribute to latency, yet with no DNA lesions occurring during the latent state.

## CNS Inflammation Induced by HSV-1

### Role of Toll-Like Receptors in Brain Inflammation by HSV-1

TLRs are components of the immune system that contribute at providing the host a first line of defense against viral infections (Xagorari and Chlichlia, 2008). However, while several TLRs have been associated with viral control and clearance, brain inflammation may be triggered by the activation of TLRs in response to HSV reactivations occurring in this tissue in a subclinical form (Kurt-Jones et al., 2004). For instance, TLR-2 and TLR-4 activation were reported during HSV-1 infection of astrocytes, with subsequent IFN type-I expression and up-regulation of the pro-inflammatory cytokine IL-6, which was dependent of viral replication (Villalba et al., 2012). Furthermore, overregulated TLR2 and TLR4 responses in PBMCs has been reported in AD patients (Zhang et al., 2012) and that continuous TLR2 activation contributes to the neuroinflammation process (McDonald et al., 2016). Also, Aβ-protein aggregates have been reported to mimic damage-associated molecular patterns (DAMPs), promoting the generation of a pro-inflammatory environment in microglia cells through TLR4 activation (Walter et al., 2007). Although this response is responsible for Aβ clearance, a sustained pro-inflammatory environmental could be detrimental for the host (Heneka et al., 2013; Chen L. et al., 2016; Go et al., 2016). Additionally, TLR2 and TLR7 responses are over-stimulated in PBMCs in MS patients when compared with healthy controls (Hamid et al., 2016; Fujiwara et al., 2018).

**Figure 3:**
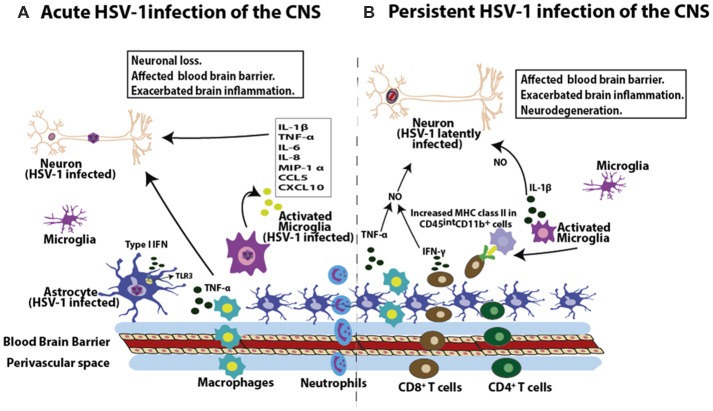
HSV-1 induces inflammation in the brain. **(A)** During acute infection of the brain, HSV-1 leads to the infiltration of macrophages and neutrophils. Moreover, HSV-1 infection induces the expression of pro-inflammatory molecules in this tissue, such as interleukin-1β (IL-1β), tumor necrosis factor-α (TNF-α), IL-6, IL-8, macrophage inflammatory protein 1-α (MIP-1α), chemokine (C-C motif) ligand 5 (CCL5) and chemokine CXCL10 by microglial cells, as well as TNF-α production by macrophages. Astrocytes in turn produce type-I interferon (IFN) mediated by TLR3 engagement in response to HSV-1. These soluble molecules will affect the permeability properties of the blood brain barrier (BBB) and potentially exacerbate brain inflammation, potentially leading to neuron insult. **(B)** HSV-1 latent CNS infection is characterized by the infiltration of CD8^+^ and CD4^+^ T cells. Importantly, these T cells are localized near latently infected neurons and are detected in a 3:1 ratio (CD8^+^ to CD4^+^ T cells). Moreover, CD8^+^ T cells can secrete IFN-γ. Prolonged microglial activation in the brain by HSV-1 infection produces increased MHC-II expression in CD45^int^CD11b^+^ cells, which lasts up to 30 days post-infection. As a consequence of immune cell infiltration into the brain during both, acute and persistent HSV-1 infection of the brain, cytokines such as TNF-α and IL-1β can affect the BBB, which can exacerbate brain inflammation. Importantly, synergistic effects between TNF-α and IFN-γ can lead to increased nitric oxide-induced neurodegeneration and demyelination in the brain.

On the other hand, although some TLRs have been shown to have detrimental effects in brain inflammation, other studies have reported that pattern recognition receptors, such as TLR3 can have important functions in ameliorating the progression of MS (Marta, 2009). In the EAE mouse model, TLR3 and TLR9 responses were found to be downregulated (Marta, 2009), and an activator of TLR3 rescued cellular infiltration in neuronal tissue and reduced demyelination (Evangelista et al., 2016; Dias et al., 2018). Interestingly, TLR3 has gained special attention because of its role in the control of HSV-1 infection of the CNS. TLR3 is located in intracellular compartments and has the capacity to sense double-stranded viral RNA (dsRNA), which activates type-I IFN signaling pathways and the production of cytokines (Figure 3A; Okun et al., 2011). TLR3 has been reported to act as a genetic factor associated with HSE, both as a dominant and recessive autosomal gene (Zhang et al., 2013). Importantly, its expression has been found to be increased in the CNS of human brains with HSE, as well as in those with neurodegenerative diseases (Jackson et al., 2006). In contrast, deficiencies in TLR3 function have been reported in children and young individuals that develop HSE during childhood (Zhang et al., 2007; Guo et al., 2011). Furthermore, an autosomal recessive deficiency in the intracellular protein UNC-93B, as well as different heterozygous mutations in TBK1, the TNFR-associated factor 3 (TRAF3), IFN regulatory factor 3 (IRF3), TYK2, MAVS, TRIF and STAT have been reported to act as factors involved in TLR3 signaling and the activation of IFN responses, and are classified as genetic etiologic mutations associated to HSE (Casrouge et al., 2006; Pérez de Diego et al., 2010; Herman et al., 2012; Mørk et al., 2015).

*In vitro* studies with induced pluripotent stem cells (iPSC)-derived cortical and trigeminal neurons that contain mutations in TLR3 pathways, described that these cells are highly susceptible to HSV-1, due to impaired TLR3-IFN immunity (Zimmer et al., 2018). However, TG neurons that were pre-stimulated with IFN-β developed an anti-HSV-1 state in TLR3-deficient trigeminal neurons, an effect that was not observed in cortical neurons, thus showing different roles for TLR3 in the CNS and peripheral nervous system during infection with HSV-1 (Lafaille et al., 2012). A recent study reported that HSV-1-infected microglia conferred a STING-dependent antiviral state to neurons and primed type-I IFN production in astrocytes through a TLR3 pathway (Reinert et al., 2016). On the other hand, a recent study reported that TLR3 is necessary for inducing innate immune responses against HSV-1 in neurons as well as astrocytes, but not in microglia (Sato et al., 2018). Interestingly, this process was mediated by TLR3 recruitment to the metabolic checkpoint kinase complex mTORC2, which induces chemokine production and TLR3 trafficking to the cell periphery thanks to the RAb7 GTPase, a protein involved in intracellular traffic processes (Sato et al., 2018).

Taken together, TLR responses have both beneficial and deleterious effects over HSV-1 infection and some neurodegenerative disorders. On one hand, alterations in TLR signaling pathways by HSV-1 infection may increase neurodegeneration processes already present in some neurodegenerative diseases. On the other hand, patients with neurodegenerative disorders that have alterations in TLRs responses might be unable to control viral infections and thus, would be more susceptible to viral reactivations than otherwise healthy individuals which could be associated with relapses in MS or worsening prognosis in AD. Nevertheless, these findings suggest that these pathologies could be treated with TLR-modulating molecules. Interestingly, recent studies have shown that the administration of TLR2, TLR4 and TLR9 antagonists may have positive effects in AD and MS (Gambuzza et al., 2014; Gooshe et al., 2014). Furthermore, TLR3 agonists could also be used as approaches for dampening the pathogenesis of patients suffering from such diseases (Boivin et al., 2008; Gambuzza et al., 2015). Hence, adequate targeting of TLR pathways could potentially reduce inflammatory processes both, in the context of HSV-1 infection and neurodegenerative disorders.

### HSV-1 Produces Immune Cell-Mediated Neuroinflammation

Importantly, HSV-1 can trigger proinflammatory responses by several cell types that it infects in the CNS, both *in vitro* and *in vivo* (Gnann and Whitley, 2017). Importantly, non-productive HSV-1 infections can also lead to the expression of cytokines and pro-inflammatory molecules, such as interleukin-1β (IL-1β), TNF-*α*, IL-6, IL-8, macrophage inflammatory protein 1*α* (MIP-1*α*), chemokine (C-C motif) ligand 5 (CCL5) and chemokine CXCL10 in human microglial cells (Lokensgard et al., 2001). Additionally, HSV-1 brain infections have been reported to display neuroimmune responses which persist in the absence of detectable virus replication (Conrady et al., 2010).

Early during HSE, the immune response in the brain is dominated by the influx of macrophages and neutrophils, which play critical roles in viral clearance (Figure 3A; Marques et al., 2008b; Terry et al., 2012). Notably, infiltrating macrophage populations have been shown to be the major source of TNF-α and microglial cells express high levels of IL-1β (Figures 3A,B; Fields et al., 2006). These two cytokines are involved in the upregulation of endothelial cell adhesion molecules, which likely affect the blood-brain barrier (BBB), which could exacerbate brain inflammation (Weiser et al., 2007). In addition, T lymphocytes have been reported to be a predominant leukocyte cell type infiltrating the brain at 14 days post-infection and is composed largely by CD8^+^ T cells that persist in this tissue up to 30 days post-infection in mice without detectable infectious virus or virus replication products (Marques et al., 2008b; Terry et al., 2012). Importantly, infiltrating CD8^+^ T cells express IFN-γ which is known to synergize with TNF-α to increase NO-induced neurodegeneration and demyelination in the brains of mice (Blais and Rivest, 2004). Such infiltrating T cells likely recognize the HSV-1 immunodominant epitope gB_498–505_, derived from the viral glycoprotein B (gB; St Leger et al., 2011) and may mediate the death of these cells during acute infection or neuroinflammation during viral latency (Chevalier et al., 2011). Interestingly, MHC-I expression in neurons has been reported to play a positive role in the development of synaptic plasticity and axonal regeneration (Cullheim and Thams, 2010), although other studies suggest that constitutive expression of MHC-I in neurons may be involved in neurodegenerative disorders by enhancing T cell-mediated CNS degeneration (Cebrián et al., 2014).

On the other hand, persisting lymphocytic cell infiltrations and elevated levels of cytokine transcripts (IFN-γ, TNF-α), as well as chemokines (CXCL10, CCL5) have been reported in human TG (Theil et al., 2003). In this regard, it has been hypothesized that this process may be the consequence of low-level expression of IE and E viral genes during latency (Du et al., 2011). Concomitantly, HSV-1 reactivation from its latent phase was demonstrated by the detection of viral ICP4 protein in the TG and cerebral cortex of mice 60 days post-infection, and was accompanied by the up-regulation of markers of neuroinflammation, such as TLR4, IFN *α*/β, and phosphorylated IRF3 (p-IRF3; Martin et al., 2014a). In line with CNS inflammation, prolonged microglial activation has also been reported in the brains of mice latently-infected with HSV, as indicated by high MHC class-II expression levels in CD45^int^CD11b^+^ cells up to 30 days post-infection (Figure 3B; Marques et al., 2008b).

Notably, several studies indicate that cytokines and chemokines could be involved in the pathology of multiple diseases associated to neurodegeneration (Reale, 2015). In AD high levels of pro-inflammatory cytokines, such as TNF-α, IL 1β and IL-6 in cerebrospinal fluid and peripheral blood of patients are expressed (Iarlori et al., 2005; Latta et al., 2015). Cytokines have also been found to be expressed near amyloid peptide deposits in post-mortem tissues (Gomez-Nicola and Boche, 2015). Likewise, immune factors seem to play a key role in MS, with IFN-γ and TNF-α secreted by brain-infiltrating T cells associated to axonal injury and elevated levels of these cytokines in peripheral blood samples of patients undergoing symptomatic relapses (Muller et al., 2004; Dendrou et al., 2005; Reale et al., 2012). Taken together, these findings suggest that HSV-1-induced expression of inflammatory factors, together with an immune-activated state in the brain could contribute to the onset or exacerbation of neuron demyelination or neurodegenerative diseases.

## Evidence Regarding a Role of HSV-1 in Neurodegenerative Diseases

### Multiple Sclerosis

MS is an autoimmune inflammatory disorder of the brain and spinal cord in which multifocal autoreactive lymphocytic infiltration leads to damage of myelin and axons (Compston and Coles, 2008). Such damage disrupts the ability of neurons to transmit nerve impulses, resulting in a widespread of disease manifestation and numerous symptoms including physical, sensorial, cognitive, and sometimes psychiatric problems (Miller et al., 2005). The last stage of the disease is associated with a widespread degeneration of the white and gray matter, resulting in brain atrophy (Dendrou et al., 2005). Although, the etiology of MS is still unknown the development of the disease is associated with an interplay between the immune system and environmental factors, including viral infections in genetically susceptible individuals (Beecham et al., 2013). Herpesviruses have long been mentioned as potential candidate viruses that could cause or enhance MS (Virtanen and Jacobson, 2012; Leibovitch et al., 2018). Importantly, several clinical studies have highlighted an association between HSV-1 and MS. The discovery of HSV-1 genetic material in tissue samples, body fluids or blood cells of patients with MS has given space for this plausible hypothesis. HSV-1 was isolated from the cerebrospinal fluid of a patient during a first episode of MS (Bergström et al., 1989). Before that, HSV-1 had been isolated from the brain of a patient with MS (Gudnadottir et al., 1964). More recently, a case-control study evaluated the prevalence of HSV-1 in PBMCs of patients with relapsing-remitting MS (RRMS) comparing it with that of healthy controls. Noteworthy, HSV-DNA tested positive in 45.1% of patients with MS and 3.4% of healthy subjects (Najafi et al., 2016). Another study also indicated that HSV-1 reactivates in the peripheral blood of patients with MS during clinical acute episodes and probably plays a role in triggering MS relapses (Ferrante et al., 2000). Finally, an increased presence of HSV-1 DNA has been reported in postmortem MS brain tissues, as compared to a control group and HSV-DNA was found in more active plaques than inactive plaques in brain tissue biopsies (Sanders et al., 1996).

On the other hand, HSV-1 seropositivity has been associated with increased risk of suffering MS in individuals that do not have the DRB1*15 allele, or otherwise low-risk individuals that have the DRB1*15 allele (Waubant et al., 2011). These findings support the possibility that HSV-1 may play a relevant role in the development of MS in individuals with specific genotypes. Likewise, mice infected with HSV-1 may or may not develop demyelination depending on the mouse and virus strain assessed (Kastrukoff et al., 2012). Studies using a recombinant HSV-1 expressing IL-2 reported the presence of auto-reactive T cells in the brain and CNS demyelination, supporting the hypothesis that both, virus and IL-2 play roles in the demyelination process and that HSV-1 could be important at initiating the destruction of myelin in the presence of elevated levels of IL-2 (Osorio et al., 2005; Zandian et al., 2011; Mott et al., 2013). Gene and environment interactions could also influence the outcome of HSV-1 infection. A recent study showed that the HSV-1 host/pathogen interactome is highly concentrated in susceptibility genes for neurological disorders, primarily for MS, with enrichment values at 4-fold, suggesting that HSV-1 may contribute to several diseases in a gene-dependent manner by modulating essential pathways involved in the onset or severity of this neurodegenerative disease (Carter, 2017).

On the other hand, microorganisms may also contribute to the pathogenesis of MS by inducing the activation and clonal expansion of self-reactive lymphocytes through molecular mimicry (Wucherpfennig and Strominger, 1995). Moreover, the Hy.1B11 T cell receptor (TCR) originated from a patient with MS showed cross-reactivity with a peptide derived from HSV-1 (UL15_154–166_), with a similar binding topology as the human myelin basic protein peptide (Sethi et al., 2013).

Although, HSE has also been reported to be a trigger of brain autoimmunity by detecting anti-N-methyl-D-aspartate (anti-NMDAR) antibodies in some patients after HSE disease (Armangue et al., 2014), the potential relationship between HSV-1 and autoimmunity in MS patients has been largely discussed and remains controversial, as some studies have shown contradictory results (Koros et al., 2014; Sotelo et al., 2014). Moreover, it is unknown whether HSV-1 brain infection could either initiate, enhance the progression or be a consequence of MS disease. In support with this latter statement, several studies have reported reduced percentages of CD8^+^ T cells in peripheral blood of MS patients, which could be associated with impaired responses against viral infections (Thompson et al., 1986; Pender et al., 2012). Additionally, a recent study showed that EBV-specific CD8^+^ T cells in individuals suffering MS displayed limited cytokine production, evidencing an exhaustion-like phenotype (Pender et al., 2017). Moreover, others found that CD8^+^ CD57^+^ T cells had increased expression of inhibitor PD-1 on the cell surface in patients with MS, as compared to healthy individuals and was associated with a negative regulation of cytotoxic responses against EBV (Cencioni et al., 2017). Hence, it is possible that defective T cell control of HSV-1 infection in MS and exhaustion of T cells in patients with MS may lead to HSV-1 reactivation in these patients, although further studies are needed to test this hypothesis.

### Alzheimer’s Disease

AD is an inflammatory neurodegenerative disease characterized by cognitive damage leading to dementia (Vinters, 2015). AD develops with pathological features including the formation of senile plaques and neurofibrillary tangles (NFTs; Vinters, 2015). Senile plaques are formed by the accumulation of Aβ, mainly Aβ_1–42_ and Aβ_1–40_, which are produced by the cleavage of the neuronal Aβ precursor protein (AβPP) and is dependent on β- and γ-secretases (Bitan et al., 2003). On the other hand, NFTs are composed of hyperphosphorylated tau proteins (Yang and Wang, 2018). Glycogen synthase kinase-3β (GSK3β) and protein kinase A (PKA) have been shown to be involved in the phosphorylation of tau proteins, which are in turn important in microtubule assembly and the synaptic plasticity and function of neurons (Kolarova et al., 2012). However, when tau proteins are hyperphosphorylated, it has been suggested that they may produce microtubule destabilization, synaptic injury and neurodegeneration (Lerchundi et al., 2011). Although the exact mechanism leading to this outcome is undetermined, gene susceptibility and brain infection by several microorganisms has been associated with the pathogenesis of AD, such as for *Chlamydophila pneumonie* (Gérard et al., 2006), *Borrelia burgdorferi* (Miklossy, 2011) and HSV-1 (Itzhaki, 2014, 2016), among others (Lurain et al., 2013; Carbone et al., 2014), which could be associated with their ability to cause chronic infections in the host. For instance, HSV-1 DNA has been detected in brain samples and found to co-localize with Aβ, being more frequently detected in the brains of AD patients than healthy controls (72% vs. 24%, respectively; Wozniak et al., 2009). Furthermore, virus reactivation may play an important role in the development of AD, as evidenced by the presence of anti-HSV-1 IgM antibodies in most people suffering from AD (Lövheim et al., 2014). Notably, the regions of the CNS damaged during HSE are related to the limbic system, in turn associated with memory and cognitive processes, which relates to AD with similar patterns of plaque distribution, supporting an association between HSV-1 brain infection and AD (Armien et al., 2010; Piacentini et al., 2014). On the other hand, HSV-1 has been found in the brains of individuals carrying the type 4 allele of the apolipoprotein E gen, suggesting that this is a susceptibility factor in AD (Lin et al., 1995). Noteworthy, patients with this allele and HSV-1 infection in the brain display an increased risk of suffering dementia, supporting a role for HSV-1 in AD (Itzhaki et al., 1997).

Importantly, HSV-1 produces the accumulation of Aβ_1–42_ and Aβ_1–40_, as well as AβPP reduction in human cultured neuronal cells *in vitro*, which was related to the up-regulation of β- and γ-secretase components in those cells (Wozniak et al., 2007). Similar results have been shown in HSV-1-infected rat cortical neurons (De Chiara et al., 2010). Interestingly, HSV-1 capsid has been shown to interact with AβPP leading to abnormal distribution of this protein in infected cells (Cheng et al., 2011). This study showed co-localization of VP26-GFP-labeled viral particles with AβPP in the cytoplasm of epithelial and neuronal cells infected with HSV-1, which allowed faster transportation of viral capsids along neurons (Cheng et al., 2011). Another study demonstrated that gB of HSV-1 shares 67% homology with the carboxyl terminal region of Aβ_1–42_, and that a synthetic peptide of gB is able to self-assemble into β-sheets with similar conformation to Aβ and produce accumulation of neurotoxic Aβ fibrils (Cribbs et al., 2000). Additionally, HSV-1 has been shown to produce calcium-dependent GSK3β activation, which translated into hyper-phosphorylation of tau and AβPP proteins, as well as the accumulation of Aβ with subsequent reductions in the activity of CREB-producing neurodegeneration associated to neuronal injury (Piacentini et al., 2015).

Recently, new evidence supporting an infection-related hypothesis for AD has been reported. It has been suggested that Aβ accumulation in the brain may be given by the fact that this peptide acts as an antimicrobial peptide (AMP) related to innate immunity (Fulop et al., 2018). Interestingly, Soscia et al., 2010 were the first to show an antibacterial and antifungal activity for Aβ peptide against numerous pathogens using microdilution susceptibility tests *in vitro*. Furthermore, they reported that brain homogenates from AD patients had higher antimicrobial activity than brains from non-AD individuals. Importantly, antiviral activity for Aβ, particularly against the influenza virus has also been reported (White et al., 2014), and that Aβ_1–42_ has greater antiviral effect than Aβ_1–40_ is consistent with a previous report that showed that Aβ_42_ had increased antibacterial effects as compared to Aβ_1–40_ (Soscia et al., 2010; White et al., 2014). More recently, another study reported that Aβ can prevent infection by HSV-1 in fibroblasts, epithelial and neuronal cells (Bourgade et al., 2014), and a study using transgenic 5XFAD mice showed that Aβ peptides can protect the host against brain infections with *Salmonella* *enterica* serovar Typhimurium, HSV-1 and HHV-6 (Kumar et al., 2016; Eimer et al., 2018). Taken together, these findings suggest that subclinical reactivation of HSV-1 in the brain of patients with AD may promote increased deposition of Aβ in this tissue, thus accelerating disease progression.

## Concluding Remarks

The high prevalence of HSV-1 infection in humans from all over the world and somewhat the low frequency of neurodegenerative diseases in the population (which is nevertheless in steady growth), have likely obscured a possible relationship between infection with this virus and neurodegenerative pathologies. However, the fact that HSV-1 can reach the brain by several mechanisms and modulate numerous key cellular processes, such as apoptosis, autophagy and cellular oxidation suggest that neuron infection with this virus can lead to brain damage because of direct damage to its cells. Furthermore, CNS damage is likely favored by the inflammation of the brain and the secretion of numerous immune-modulatory cytokines in this tissue. Importantly, some studies provide compelling data that suggest close ties between HSV-1 infection of the brain and neurodegenerative diseases, which would not be surprising given that other herpesviruses have recently been associated with MS. These findings call for further studies that corroborate this possible relationship and evaluate the interrelationship between HSV-1 and neurodegeneration, assessing for example how neurodegeneration affects viral reactivation in the brain and the corresponding underlying molecular mechanisms. Animal models that undergo HSV-1 infections that recapitulate the manifestations of disease in humans, with somewhat similar neurodegenerative disease aspects would be of great utility. Interestingly, recent studies with tree shrews (*Tupaia belangeri*), which are animals that share genomic and transcriptomic similarities with humans indicate that they may be valuable for such studies, as they have been reported to display HSV-1-related diseased manifestations that are similar with those seen in humans (i.e., latent infections and reactivations) and to suffer neurodegenerative disorders that may model AD and multiple sclerosis (Li et al., 2016; Xiao et al., 2017).

## Author Contributions

LD, MF, DÁ, SB, CR and PG wrote the manuscript and designed the figures. All authors reviewed the manuscript.

## Conflict of Interest Statement

The authors declare that the research was conducted in the absence of any commercial or financial relationships that could be construed as a potential conflict of interest.
